# Changes in Impaired Fasting Glucose and Borderline High Low-Density Lipoprotein-Cholesterol Status Alter the Risk of Cardiovascular Disease: A 9-Year Prospective Cohort Study

**DOI:** 10.3389/fcvm.2022.882984

**Published:** 2022-06-21

**Authors:** Xianxuan Wang, Yan-Feng Zhou, Zegui Huang, Xinran Yu, Zekai Chen, Zefeng Cai, Yulong Lan, Werijian Li, Zhiwei Cai, Wei Fang, Guanzhi Chen, Weiqiang Wu, Shouling Wu, Youren Chen

**Affiliations:** ^1^Second Clinical College, Shantou University Medical College, Shantou, China; ^2^Department of Cardiology, Second Affiliated Hospital of Shantou University Medical College, Shantou, China; ^3^Department of Epidemiology and Biostatistics, School of Public Health, Tongji Medical College, Huazhong University of Science and Technology, Wuhan, China; ^4^Department of Anesthesiology, North China University of Science and Technology, Tangshan, China; ^5^Department of Epidemiology, University Medical Center Groningen, University of Groningen, Groningen, Netherlands; ^6^Second Clinical College, China Medical University, Shenyang, China; ^7^Department of Cardiology, Kailuan General Hospital, Tangshan, China

**Keywords:** impaired fasting glucose, borderline high low-density lipoprotein-cholesterol, cardiovascular disease, dynamic changes, cohort study

## Abstract

**Background:**

We aimed to characterize the relationships of the changes in impaired fasting glucose (IFG) and borderline high low-density lipoprotein-cholesterol (LDL-C) status with cardiovascular disease (CVD).

**Methods:**

A total of 36,537 participants who did not have previous CVD, diabetes mellitus, or high LDL-C (≥ 4.1 mmol/L), nor were taking lipid-lowering drugs were recruited from the Kailuan study. The participants were allocated to six groups according to their baseline and follow-up fasting blood glucose (FBG) and LDL-C concentrations: (1) both were normal; (2) both normal at baseline, one abnormality subsequently; (3) both normal at baseline, both abnormal subsequently; (4) at least one abnormality that became normal; (5) at least one abnormality at baseline, a single abnormality subsequently; and (6) at least one abnormality, two abnormalities subsequently. The outcomes were CVD and subtypes of CVD (myocardial infarction and stroke). Multiple Cox regression models were used to calculate adjusted hazard ratio (HR) and confidence interval (95% CI).

**Results:**

During a median follow-up period of 9.00 years, 1,753 participants experienced a CVD event. After adjustment for covariates, participants with IFG in combination with a borderline high LDL-C status at baseline and follow-up had higher risks of CVD (HR: 1.52; 95% CI: 1.04–2.23 and HR: 1.38, 95% CI: 1.13–1.70, respectively) compared with those with normal fasting blood glucose and LDL-C. Compared with participants that remained normal, those who changed from normality to having two abnormalities were at a higher risk of CVD (HR: 1.26; 95% CI: 0.98–1.61), as were those who changed from at least one abnormality to two abnormalities (HR: 1.48, 95% CI: 1.02–2.15).

**Conclusion:**

Changes in IFG and borderline high LDL-C status alter the risk of CVD and its subtype, implying that it is important to focus on such individuals for the prevention and control of CVD.

## Introduction

Cardiovascular disease (CVD) is a major cause of death and disability worldwide (1). The global number of deaths from CVD was approximately 17.9 million in 2019, accounting for 32% of all deaths (2). There is growing evidence that disorders of glucolipid metabolism, especially diabetes mellitus and high low-density lipoprotein-cholesterol (LDL-C) concentration, are independent risk factors for CVD (3, 4); and previous studies have shown that impaired fasting glucose [IFG, the criteria of the American Diabetes Association, fasting blood glucose (FBG) 5.6-6.9 mmol/L] and borderline high LDL-C status is associated with a higher risk of CVD, despite not meeting the diagnostic criteria for diabetes mellitus or high LDL-C status (5–7). Several large scale meta-analyses (8, 9) found that the risks of CVD, heart failure were 1.13- and 1.09- fold higher in those with IFG compared to those with FBG; and in the Framingham study, the risks of CVD were 1.50- and 1.85-fold higher in men and women with borderline high LDL-C, respectively, compared to those with normal LDL-C (7).

However, the presence of a single risk factor is rare (10, 11), and we hypothesized that people with multiple risk factors would be at a higher risk of CVD. Nevertheless, there have been relatively few studies of the combined effects of risk factors on the prevalence of CVD. The Asia Pacific Cohort Studies Collaboration found a 6.84-fold higher risk of CVD in individuals in the upper quartiles of both total cholesterol concentration and systolic blood pressure, compared to those in the lowest quartiles (12). IFG and borderline high LDL-C status are independent risk factors for CVD, and often coexist and interact (10, 11). Therefore, we hypothesized that there may be a combined effect of IFG and borderline high LDL-C status on the risk of CVD.

In addition, cardiovascular risk factors may change over time. Most previous studies that linked IFG and borderline high LDL-C status with CVD have been conducted at a single time point, but FBG and LDL-C are affected by many factors, including age, diet, and medication (13, 14). Therefore, a single measurement does not fully reflect the relationship of IFG and borderline high LDL-C status with CVD, and multiple measurements are likely to provide more accurate assessments. Furthermore, serial high measured concentrations are more likely to be of prognostic value than a single measurement. For example, Kabootari et al. (15) found a 28% lower risk of CVD in individuals who progressed to IFG compared with those who had persistently normal FBG. In addition, Jeong et al. (16) found that the risk of CVD in individuals with normal total cholesterol who subsequently developed borderline high concentrations (180–240 mg/dL) was 1.05-fold higher than in those with persistently normal total cholesterol concentration (< 180 mg/dL).

Thus, most previous studies were conducted in European and American populations and focused on the effects of changes in single parameters on health-related outcomes, whereas there have been few studies of the relationships between multiple changes in glucose and lipid concentrations. In the present study, we aimed to characterize the relationships of changes in IFG and borderline high LDL-C status with CVD.

## Materials and Methods

### Study Sample

The data were derived from the Kailuan study (trial registration number: ChiCTR-TNRC-11001489), which is an ongoing prospective cohort study conducted in Tangshan, China. Details of the study design and procedures have been provided previously (17, 18). In brief, a total of 101,510 participants (81,110 men and 20,400 women) agreed to take part in and complete the first examinations during 2006–2007. Follow-up evaluations included biennial measurements of laboratory parameters and occurrence of adverse events; and six follow-up visits (2008–2009, 2010–2011, 2012–2013, 2014–2015, 2016–2017, and 2018–2019) have been completed so far.

**FIGURE 1:**
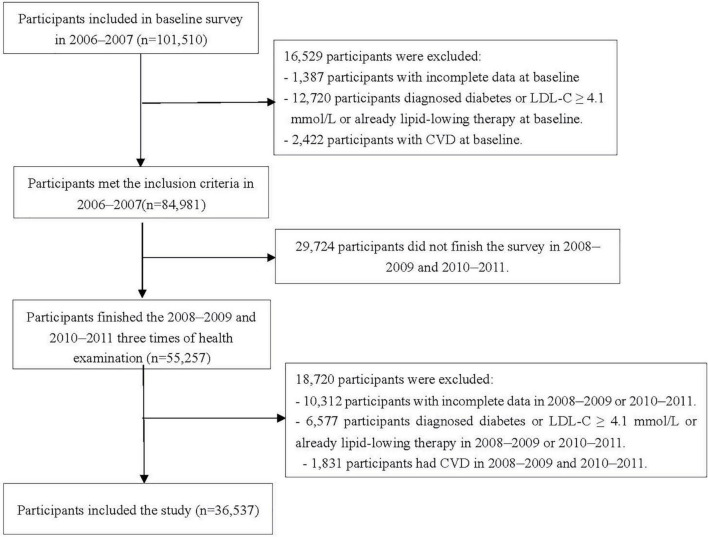
Flowchart of this study.

In the study, changes in IFG and borderline high LDL-C status were determined from January 2006 to December 2011. The first survey (2006–2007) was set as baseline survey, and the 2010–2011 survey was set as the start time-point of follow-up, given the events occurred after the changes. The timeline of this study is shown in Supplementary Figure 1. We excluded 1,387 and 10,312 participants who had missing key data (e.g., FBG and LDL-C concentrations) needed to identify their IFG and borderline high LDL-C status in or before 2010; 12,720 and 6,577 participants diagnosed diabetes or LDL-C ≥ 4.1mmol/l or already lipid-lowering therapy in or before 2010; 2,422 and 1,831 participants with the history of CVD in or before 2010; 29,724 participants who did not undergo three examinations during 2006–2007, 2008–2009, and 2010–2011. Finally, data from 36,537 participants were included (Figure 1).

The study protocol was approved by the ethics committee of Kailuan General Hospital (approval number: 2006-05). All the participants provided their written informed consent.

### Changes in Impaired Fasting Glucose and Low-Density Lipoprotein-Cholesterol Status

According to the follow-up report on the diagnosis of diabetes mellitus (19), FBG status was classified as normal (FBG < 5.6 mmol/L, without the use of glucose-lowering drugs), IFG (5.6 mmol/L ≤ FBG < 6.9 mmol/L, without the use of glucose-lowering drugs), or diabetes (FBG ≥ 7.0 mmol/L or the use of antidiabetic medication). According to the 2016 Chinese guideline for the management of dyslipidemia in adults (20), LDL-C status was classified as normal (< 3.4 mmol/L without the use of lipid-lowering drugs), borderline high (3.4 mmol/L ≤ LDL-C < 4.0 mmol/L, without the use of lipid-lowering drugs), or high (LDL-C ≥ 4.1 mmol/L or the use of lipid-lowering drugs) (21).

To explore the combined effect of IFG and borderline high LDL-C at baseline and follow-up, the participants were placed into four groups: normal FBG and LDL-C, IFG and normal LDL-C, normal FBG and borderline high LDL-C, and IFG and borderline high LDL-C. In addition, to characterize the relationships of the changes in IFG and borderline high LDL-C status between baseline and follow-up and the risk of CVD, the participants were placed into six groups: (1) normal concentrations at both time points; (2) normal concentrations changing to a single abnormality (IFG or borderline high LDL-C); (3) normal concentrations changing to two abnormalities (IFG and borderline high LDL-C); (4) at least one abnormality (IFG and/or borderline high LDL-C) changing to normal; (5) at least one abnormality (IFG and/or borderline high LDL-C) changing to a single abnormality (IFG or borderline high LDL-C); or (6) at least one abnormality (IFG and/or borderline high LDL-C), changing to two abnormalities (IFG and borderline high LDL-C).

### Outcomes

The CVD outcomes were defined as myocardial infarction (MI) or any type of stroke (hemorrhagic or ischemic). We used ICD-10th revision codes to identify CVD cases (I21 for MI, I63 for ischemic stroke, and I60 to I61 for hemorrhagic stroke) (22). The databases of CVD diagnoses were obtained from the Municipal Social Insurance Institution and the Hospital Discharge Register. These were updated annually during the follow-up period. An expert panel, including three clinical physicians who have obtained the qualification of clinical physician of the People’s Republic of China, independently collected and reviewed the annual discharge records from the 11 participating hospitals to identify patients who were suspected of having CVD. The disagreements of the two researchers were resolved by another researcher (23, 24). A diagnosis of MI was made on the basis of the symptoms, electrocardiographic findings, and changes in myocardial enzyme activities, according to the World Health Organization’s Multinational Monitoring of Trends and Determinants in Cardiovascular Disease criteria (25). Stroke was diagnosed on the basis of neurological signs, symptoms, and the findings of neuroimaging, including computed tomography and magnetic resonance imaging, according to the World Health Organization’s criteria (26). For those who experienced two or more events, the time and nature of the first event was recorded as the endpoint, and those who did not experience an event underwent their last follow-up examination on December 31, 2019.

### Covariates

Other related data were collected via self-reported questionnaires (including age, sex, educational level, smoking and drinking status, physical exercise, and anti-hypertensive medications), basic anthropometric measurements (body height, weight and blood pressure), and the laboratory test data, these were updated every 2 years, as described previously (27). Measurements of body weight, height and blood pressure were made by trained physicians using a standardized protocol. Blood pressure was measured while seated using a calibrated mercury sphygmomanometer after a 15-min rest, and the mean values of three measurements of systolic blood pressure (SBP) and diastolic blood pressure (DBP) were recorded. Body mass index (BMI) was calculated as body weight (kg) divided by height squared (m^2^). Obesity was defined as a BMI ≥ 28 kg/m^2^, overweight or above was defined as BMI ≥ 25kg/m^2^ (28–30). Current smokers were defined as someone who smoked ≥ 1 cigarette/day on average during the preceding year. Current drinkers were defined as someone who drank a mean of 100 mL of an alcoholic drink (types of wine) per day for at least the preceding year. Physical exercise was defined as performing exercise ≥ 3 times/week for ≥ 30 min each (24). Hypertension (31) was defined as either a blood pressure ≥ 140/90 mmHg, the use of an anti-hypertensive medication, or a history of hypertension.

All the participants fasted for at least 8 h and 5 mL of venous blood was obtained on the morning of each physical examination. The serum concentrations of FBG, LDL-C, high-density lipoprotein-cholesterol (HDL-C), and creatinine were measured by the hexokinase/glucose-6-phosphate dehydrogenase method, direct method-surfactant clearance method, direct method-select inhibition method, and alkaline picrate (Jaffé) reaction, respectively, using a Hitachi 7600 autoanalyzer (Hitachi, Tokyo, Japan) at the central laboratory of Kailuan General Hospital (23, 32–34). And estimated glomerular filtration rate (eGFR) which was calculated from creatinine following the CKD-EPI (Chronic Kidney Disease Epidemiology Collaboration) formula (35).

### Statistical Analysis

For the baseline characteristics of the participants, normally distributed continuous data are summarized using mean ± standard deviation and were analyzed using one-way ANOVA. Skewed continuous data are described using median and interquartile range and were analyzed using the Wilcoxon rank sum test. Categorical data are described using number and percentage and were analyzed using the chi-square test. A fully conditional specification method was used to impute missing values for covariates using the multivariate imputation by chained equation (MICE) method (36). Details of the missing covariates are presented in Supplementary Table 1. The cumulative incidence of CVD for each group was calculated using the Kaplan-Meier method and they were compared using the log-rank test. Multivariate Cox proportional hazard regression was performed to determine the hazard ratio (HR) and 95% confidence interval (CI) for incident CVD. In the Cox model for the analysis of the combined effect of IFG and borderline high LDL-C at baseline and follow-up, we adjusted for sex, age (continuous variable), obesity (categorical variable, yes or no), hypertension (categorical variable, yes or no), use of anti-hypertensive drugs (categorical variable, yes or no), smoking status (categorical variable, yes or no), drinking status (categorical variable, yes or no), exercise status (categorical variable, yes or no), educational level (categorical variable), HDL-C (continuous variable), eGFR (continuous variable). When analyzing the changes in IFG and borderline high LDL-C status, baseline serum FBG and LDL-C concentrations were further adjusted for.

In addition, stratified analyses were performed according to the age groups, sex, hypertension, anti-hypertension drugs, smoking and drinking status, and physical activity of the participants. To test the robustness of our findings, the following sensitivity analyses were performed: (1) the exclusion of individuals who developed CVD outcomes occurring within the first year of the study; (2) the exclusion of individuals missing covariable; (3) adjustment for the overweight or above instead of obesity in follow-up; (4) adjustment for covariates at baseline when analyzing the changes in IFG and borderline high LDL-C status; (5) adjustment for covariates at baseline and follow-up when analyzing the changes in IFG and borderline high LDL-C status; and (6) with categorization of the changes in IFG and borderline high LDL-C status as follows: (a) both remained normal; (b) both were normal, then at least one abnormality developed; (c) at least one abnormality, changing to normality; and (d) there was a persistent abnormality (IFG and/or borderline high LDL-C).

The study was analyzed using SAS version 9.4 (SAS Institute, Cary, NC, United States), and differences were considered statistically significant when *P* < 0.05 (two-sided).

## Results

### Baseline Characteristics

The mean age of the 36,537 participants was 56.74 ± 11.90 years and 27,841 (76.20%) were male. The prevalence of persistent normal concentrations, normality changing to a single abnormality, normality changing to two abnormalities, at least one abnormality changing to normality, at least one abnormality changing to a single abnormality, and at least one abnormality changing to two abnormalities was 57.73%, 22.76%, 2.66%, 9.87%, 6.06%, and 0.93%, respectively. Participants with at least one abnormality, changing to two abnormalities, were older, had a poorer metabolic profile (FBG, and LDL-C), and higher prevalence of obesity and hypertension than those in the other groups (*P* < 0.01; Table 1).

**TABLE 1:** Follow-up characteristics of study participants.

Baseline		Both LDL-C and FBG Normal			IFG and (or) Borderline high LDL-C			
			
Follow-up	Total	Both FBG and LDL-C Normal	IFG or Borderline high LDL-C	IFG and Borderline high LDL-C	Both FBG and LDL-C Normal	IFG or Borderline high LDL-C	IFG and Borderline high LDL-C	*P* value
Participant	36537	21091	8316	972	3605	2214	339	-
Age(years)	56.74 ± 11.90	55.63 ± 12.10	58.42 ± 11.56	60.39 ± 11.02	56.55 ± 11.64	59.15 ± 10.73	60.83 ± 10.13	<0.01
Male, N (%)	27841(76.20)	15291(72.50)	6658(80.06)	760(78.19)	2911(80.75)	1925(86.95)	296(87.32)	<0.01
BMI (kg/m^2^)	24.56 ± 3.30	24.27 ± 3.28	24.92 ± 3.28	25.74 ± 3.28	24.62 ± 3.29	25.22 ± 3.29	25.86 ± 3.06	<0.01
SBP (mmHg)	128.36 ± 18.90	125.71 ± 18.44	131.65 ± 18.53	135.93 ± 19.13	129.24 ± 18.91	134.83 ± 19.50	139.20 ± 18.45	<0.01
DBP (mmHg)	83.28 ± 10.76	82.05 ± 10.66	85.02 ± 10.67	86.66 ± 10.20	83.55 ± 10.65	85.82 ± 10.45	88.55 ± 11.66	<0.01
FBG (mmol/L)	5.18 ± 0.59	4.89 ± 0.40	5.71 ± 0.49	5.95 ± 0.31	4.96 ± 0.39	5.86 ± 0.52	6.08 ± 0.37	<0.01
LDL-C (mmol/L)	2.44 ± 0.68	2.27 ± 0.59	2.64 ± 0.72	3.64 ± 0.20	2.38 ± 0.58	2.66 ± 0.71	3.64 ± 0.19	<0.01
HDL-C (mmol/L)	1.53 ± 0.39	1.52 ± 0.39	1.54 ± 0.39	1.56 ± 0.38	1.50 ± 0.37	1.53 ± 0.38	1.57 ± 0.37	<0.01
eGFR(ml/min/1.73m^2^)	89.73 ± 20.02	90.63 ± 20.53	88.08 ± 19.31	85.82 ± 18.94	89.50 ± 19.52	89.93 ± 18.71	86.28 ± 17.72	<0.01
Obesity, N (%)	5202(14.24)	2626(12.45)	1371(16.49)	218(22.43)	485(13.45)	418(18.88)	84(24.78)	<0.01
Current smoker, N (%)	12232(33.48)	6790(32.19)	2921(35.13)	347(35.70)	1207(33.48)	840(37.94)	127(37.46)	<0.01
Current drinker, N (%)	12966(35.49)	7051(33.43)	3072(36.94)	406(41.77)	1334(37.00)	943(42.59)	160(47.20)	<0.01
Physical exercise*, N (%)	5240(14.34)	2719(12.89)	1294(15.56)	199(20.47)	566(15.70)	389(17.57)	73(21.53)	<0.01
Senior college, N (%)	10333(28.28)	6558(31.09)	2007(24.13)	210(21.60)	999(27.71)	484(21.86)	75(22.12)	<0.01
Hypertension, N (%)	13092(35.83)	6789(32.19)	3451(41.50)	439(45.16)	1289(35.76)	970(43.81)	154(45.43)	<0.01
Anti-hypertension drugs, N (%)	4170(11.41)	2104(9.98)	1083(13.02)	159(16.36)	419(11.63)	355(16.03)	50(14.75)	<0.01

*Being physical exercise was defined as performing exercise ≥ 3 times/week for ≥ 30 min each.

*IFG, impaired fasting glucose; BMI, body mass index; SBP, systolic blood pressure; DBP, diastolic blood pressure; FBG, fasting blood glucose; HDL-C, high-density lipoprotein-cholesterol; LDL-C, low-density lipoprotein-cholesterol; eGFR: estimated glomerular filtration rate.*

### Cumulative Incidence of Cardiovascular Disease

During the median follow-up period of 9.00 (interquartile range: 8.76–9.26) years, 1,753 CVD outcomes occurred, comprising 334 incident MI cases and 1,419 incident stroke cases. The cumulative incidences of CVD in the baseline normal FBG and LDL-C, IFG and normal LDL-C, normal FBG and borderline high LDL-C, and IFG and borderline high LDL-C groups were 4.74%, 6.62%, 5.71%, and 10.63%, respectively; and the cumulative incidences follow-up of CVD in the normal FBG and LDL-C, IFG and normal LDL-C, normal FBG and borderline high LDL-C, and IFG and borderline high LDL-C groups were 4.40%, 6.13%, 6.73%, and 7.98%, respectively.

The cumulative incidences of CVD in the persistent normality, normality changing to a single abnormality, normality changing to two abnormalities, at least one abnormality changing to normality, at least one abnormality changing to a single abnormality, and at least one abnormality changing to two abnormalities groups were 4.16%, 5.88%, 7.40%, 5.82%, 7.60%, and 9.65%, respectively, and these differed significantly according to the log-rank test (*P* < 0.001).

### Relationships of a Combination of Impaired Fasting Glucose and Borderline High Low-Density Lipoprotein-Cholesterol Status With Cardiovascular Disease at Baseline and Follow-Up

Table 2 shows the relationships of a combination of IFG and borderline high LDL-C status with the risk of CVD. The HR for a combination of IFG and borderline high LDL-C was highest, compared with normal FBG and LDL-C, both at baseline and follow-up (1.52, 95% CI: 1.04–2.23 and 1.38, 95% CI: 1.13–1.70, respectively). In addition, participants in IFG and borderline high LDL-C was higher risk of MI, ischemic and hemorrhagic stroke, compared with normal FBG and LDL-C, both at baseline and follow-up.

**TABLE 2:** Influence of combined effects of IFG and borderline high LDL-C on CVD.

	Baseline			Follow-up		
		
	Case/Total	IR*	HR (95%CI) ^a^	Case/Total	IR*	HR (95%CI) ^b^
**Cardiovascular disease**						
Normal FBG and LDL-C	1382/30379	5.28	1.00	1038/24696	4.86	1.00
IFG and Normal LDL-C	281/4630	7.16	1.22(1.07,1.38)	479/8484	6.63	1.07(0.96,1.20)
Normal FBG and Borderline High LDL-C	63/1197	6.21	1.01(0.78,1.29)	134/2046	7.73	1.34(1.12,1.61)
IFG and Borderline High LDL-C	27/331	9.84	1.52(1.04,2.23)	102/1311	9.33	1.38(1.13,1.70)
**Myocardial infarction**						
Normal FBG and LDL-C	259/30379	0.97	1.00	193/24696	0.89	1.00
IFG and Normal LDL -C	58/4630	1.45	1.32(1.00,1.76)	85/8484	1.15	1.04(0.80,1.34)
Normal FBG and Borderline High LDL-C	11/1197	1.06	0.92(0.50,1.69)	35/2046	1.98	1.91(1.33,2.74)
IFG and Borderline High LDL-C	6/331	2.12	1.82(1.01,4.09)	21/1311	1.86	1.56(1.00,2.47)
**Stroke**						
Normal FBG and LDL-C	1123/30379	4.27	1.00	845/24696	3.94	1.00
IFG and Normal LDL-C	223/4630	5.64	1.19(1.03,1.37)	394/8484	5.43	1.08(0.96,1.22)
Normal FBG and Borderline High LDL-C	52/1197	5.10	1.02(0.77,1.34)	99/2046	5.66	1.20(0.98,1.48)
IFG and Borderline High LDL-C	21/331	7.55	1.44(1.00,2.22)	81/1311	7.34	1.33(1.05,1.67)
**Ischemic stroke**						
Normal FBG and LDL-C	1004/30379	3.81	1.00	764/24696	3.54	1.00
IFG and Normal LDL-C	198/4630	5.00	1.20(1.04,1.39)	345/8484	4.74	1.09(0.97,1.23)
Normal FBG and Borderline High LDL-C	49/1197	4.80	1.02(0.77,1.35)	92/2046	5.24	1.21(0.98,1.49)
IFG and Borderline High LDL-C	19/331	6.83	1.46(0.98,2.26)	74/1311	6.69	1.36(1.08,1.71)
**Hemorrhagic stroke**						
Normal FBG and LDL-C	165/30379	0.62	1.00	126/24696	0.58	1.00
IFG and Normal LDL-C	32/4630	0.79	1.40(1.21,1.61)	55/8484	0.74	1.22(1.08,1.38)
Normal FBG and Borderline High LDL-C	3/1197	0.29	1.02(0.77,1.35)	10/2046	0.66	1.31(1.06,1.61)
IFG and Borderline High LDL-C	2/331	0.72	1.65(1.07,2.55)	9/1311	0.79	1.73(1.37,2.18)

*Case per 1000 person-years.

*^a^Model: adjusted for age, sex, obesity, smoking status, drinking status, physical exercise status, educational level, hypertension, use of anti-hypertensive drugs, HDL-C, eGFR in 2006.*

*^b^Model: adjusted for age, sex, obesity, smoking status, drinking status, physical exercise status, educational level, hypertension, use of anti-hypertensive drugs, HDL-C, eGFR in 2010.*

*IFG, impaired fasting glucose; FBG, fasting blood glucose; HDL-C, high-density lipoprotein-cholesterol; LDL-C, low-density lipoprotein-cholesterol; CVD, cardiovascular disease; HR, hazard ratio; CI, confidence interval; IR, incidence rate, eGFR: estimated glomerular filtration rate.*

*I63 for ischemic stroke, and I60 to I61 for hemorrhagic stroke.*

### Relationships of the Changes in Impaired Fasting Glucose and Borderline High Low-Density Lipoprotein-Cholesterol Status With Cardiovascular Disease

**FIGURE 2:**
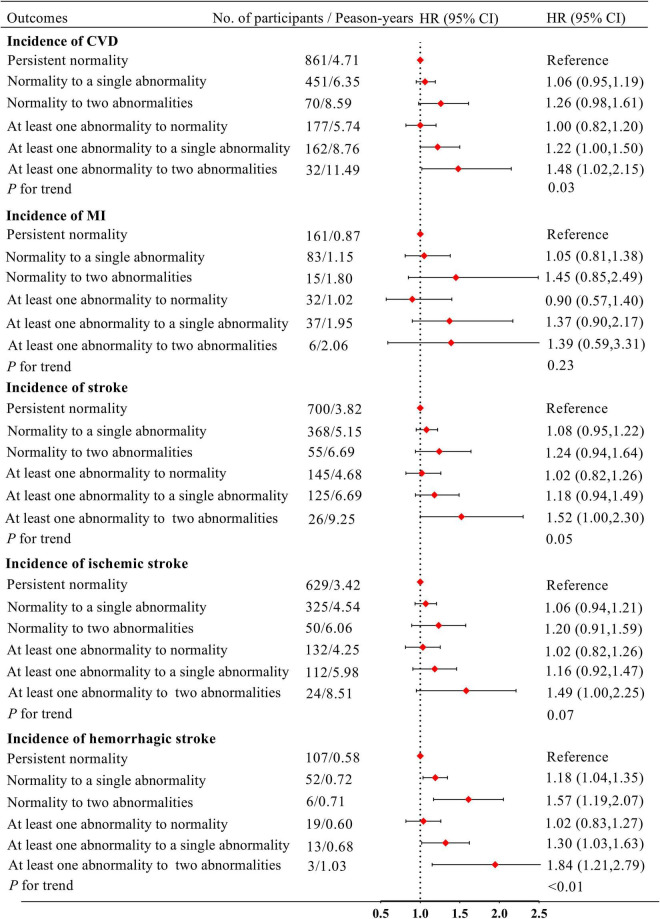
Model adjusted for age, sex, obesity, smoking status, drinking status, physical exercise status, educational level, hypertension, use of anti-hypertensive drugs, HDL-C in 2010 and FBG, LDL-C in 2006. IFG, impaired fasting glucose; FBG, fasting blood glucose; HDL-C, high-density lipoprotein-cholesterol; LDL-C, low-density lipoprotein-cholesterol; CVD, cardiovascular disease; HR, hazard ratio; CI, confidence interval.

Figure 2 shows the relationships of the changes in IFG and borderline high LDL-C status with CVD. After adjustment for covariates, participants who were initially normal but developed two abnormalities had non-significantly higher risks of CVD, MI, stroke and ischemic stroke than those who were normal throughout (HR: 1.26, 95% CI: 0.98–1.61; HR: 1.45, 95% CI: 0.85–2.49; HR: 1.24, 95% CI: 0.94–1.64 and HR: 1.20, 95% CI: 0.91–1.59, respectively). In addition, participants with at least one abnormality, changing to two abnormalities, were at significantly higher risk of CVD, MI, stroke, ischemic stroke, and hemorrhagic stroke compared with those who were normal throughout (HR: 1.48, 95% CI: 1.02–2.15; HR: 1.39, 95% CI: 0.59–3.31; HR: 1.52, 95% CI: 1.00–2.30; HR: 1.49, 95% CI: 1.00–2.25; and HR: 1.84, 95% CI: 1.21–2.79, respectively). Compared with participants who were normal throughout, there were not higher risks of CVD, MI, stroke, ischemic stroke, and hemorrhagic stroke in those who had at least one abnormality, changing to normality (HR: 1.00, 95% CI: 0.82–1.20; HR: 0.90, 95% CI: 0.57–1.40; HR: 1.02, 95% CI: 0.82–1.26; HR: 1.02, 95% CI: 0.82–1.26; and HR: 1.02, 95% CI: 0.83–1.27; respectively).

### Stratified Analyses

Although the associations were not significantly different in the aforementioned subgroups (*P* for interaction = 0.51 for hypertension status, *P* for interaction = 0.86 for antihypertensive drugs use, *P* for interaction = 0.11 for smoking status, *P* for interaction = 0.09 for drinking status, and *P* for interaction = 0.25 for physical activity), there are not association the changes in IFG and borderline high LDL-C status with CVD in those without hypertension, smoking and drinking status, physical activity. Those findings demonstrate that interactions between potential risk factors affect the incidence of CVD, MI, ischemic stroke, and hemorrhagic stroke (Supplementary Table 2).

### Sensitivity Analyses

The results of the sensitivity analyses in which participants who developed CVD the first year of the study were excluded, in which the covariates that were adjusted for were changed, in which adjusted for the overweight or above instead of obesity in follow-up, and in which the participants were regrouped were largely consistent with the results of the main analysis. Participants with normal concentrations of FBG and LDL-C who subsequently showed two abnormalities were at higher risk of CVD, but the highest risk of CVD was associated with at least one abnormality, changing to two abnormalities, versus normality throughout (Supplementary Tables 3, 4).

## Discussion

This was the first large population-based cohort study conducted in China to characterize the relationships of changes in IFG and borderline high LDL-C status with the incidences of MI, ischemic and hemorrhagic stroke. We found that IFG and borderline high LDL-C have combined effects on the incidences of CVD and its subtypes and that the risks of CVD and its subtypes are affected by changes in FBG and LDL-C status. After adjustment for covariates, participants who developed IFG and borderline high LDL-C from normality were more likely to develop CVD than those with concentrations that were normal throughout (approximately 1.26-fold). However, those with at least one abnormality that became normal did not have a higher risk of CVD than those who were normal throughout.

We have shown relationships between a combination of IFG and borderline high LDL-C with CVD. Both at baseline and subsequently, the risk of CVD in participants with IFG and borderline high LDL-C was higher than in those with normal FBG and LDL-C, and was also higher than in participants with a high concentration of only one of these substances. Although there have been few studies of the relationship of IFG in combination with borderline high LDL-C and CVD, a similar approach has been used to analyze the relationship of a combination of IFG and dyslipidemia with CVD. Hashemi et al. (37) found that the risk of CVD was 1.30-fold higher in individuals with both IFG and dyslipidemia than in those with normal FBG and lipids, which is consistent with the present findings. In addition, we found that the risk associated with a borderline high LDL-C status increased, which may be related to the pathogenesis of atherosclerosis (38, 39). And the risk associated with IFG decreased during the follow-up period, which might be explained by a change in lifestyle. This is consistent with the finding of Kabootari et al. (15), where a 28% reduction in the risk of CVD was found in individuals who progressed from normal fasting glucose to IFG or impaired glucose tolerance. Moreover, in our study we found that the IFG and borderline high LDL-C also is the risk factor for hemorrhagic stroke. Jin et al. (40) found participants with FBG concentrations 6.0 to 6.99mmol/L had a 31% higher risk of hemorrhagic stroke relative to those with the FBG 4.00 to 5.59 mmol/L. For the relationship of LDL-C with hemorrhagic stroke, previous observational studies showed inconsistent results (41–44). We found that participants in normal FBG and borderline high LDL-C have a higher risk than normal FBG and LDL-C at follow-up. We have to acknowledge that the results may be caused by insufficient sample size hemorrhagic stroke events. Taken together, these findings demonstrate that interactions between potential risk factors affect the incidence of CVD. Therefore, simultaneous interventions targeting a number of risk factors should be performed to achieve an optimal preventive effect.

We also found that changes in the status of participants with respect to IFG and borderline high LDL-C status alter the risk of CVD. The development of IFG and borderline high LDL-C from normality was associated with non-significantly higher risks of CVD and its subtypes than long-term normal concentrations (HR: 1.26, 95% CI: 0.98–1.61), perhaps because of insufficient sample size, but implying that these abnormalities may cause significant harm. Similarly, He et al. (45) found that differing patterns of changes in metabolic parameters were associated with different risks of CVD. The effects of particular risk factors on CVD depend on the intensity and duration of exposure (46), and because serum FBG and LDL-C concentrations tend to increase with age (47), the risk of CVD would be expected to be higher in individuals with IFG and borderline high LDL-C. We also found no increase in the risk of CVD in those participants who had at least one abnormality at baseline, but none subsequently (HR: 1.00, 95% CI: 0.82–1.20). Consistent with this, Jeong et al. (16) found that individuals with a high total cholesterol concentration (≥ 240 mg/dL) who subsequently had a normal concentration were at 35% lower risk of CVD. In addition, Koskinen et al. (48) found that recovery from metabolic syndrome was associated with lower carotid intima-media thickness, greater flow-mediated dilatation, and greater carotid artery distensibility. Taken together, these findings suggest that interventions targeting CVD risk factors, such as IFG, borderline high LDL-C, and metabolic syndrome, would reduce the risks of subsequent deleterious outcomes in the long term. Therefore, healthcare professionals should pay more attention to the effects of changes in IFG and borderline high LDL-C status on CVD, regularly screen individuals with IFG and borderline high LDL-C, and advise such individuals to improve their lifestyles in order to prevent CVD.

Although the mechanisms underlying the higher risk of CVD events in individuals with IFG and borderline high LDL-C remain unclear, the present findings are biologically plausible. First, disorders of glucolipid metabolism and CVD share genetic contributors. Several large genome-wide association studies have recently shown that genetic factors affecting glucolipid metabolism are also risk factors for CVD (49–52). Second, disorders of glucolipid metabolism are complex diseases caused by the interaction of genetic predisposition and unhealthy lifestyle and dietary habits. Smoking, sedentary behavior, and the consumption of a high-fat, high-sugar diet (53) are risk factors for both glucolipid disorders and CVD (3). Third, previous studies have shown that disorders of glucolipid metabolism can induce the release of pro-inflammatory factors, such as tumor necrosis factor-α and interleukin-6 (54), cause blood hypercoagulability, damage vascular endothelial cells, and participate in and promote the development of atherosclerosis (55, 56), which are key components of the pathogenesis of CVD (57). Finally, IFG and borderline high LDL-C interact with each other. Adiponectin-related abnormalities in lipid metabolism can lead to insulin resistance, which predisposes toward glucose metabolism disorders (58, 59), while conversely, glucose metabolism disorders result in greater fatty acid oxidation and glycosylation, which in turn affect lipid metabolism (60). Thus, a combination of IFG and borderline high LDL-C may create a vicious cycle that exacerbates atherosclerosis and increases the risk of CVD.

The present findings have implications for clinical and public health practice. First, the risk of CVD varies between individuals with differing FBG and LDL-C status. In the present study, approximately 30.57% of individuals with normal FBG and LDL-C progressed to IFG and (or) borderline high LDL-C, with an associated increase in their risk of CVD. In addition, we also found that the cases of stroke were much higher than MI, which is consistent with previous studies (61). Recent evidence from the China cardiovascular diseases report 2018 found that of the approximately 290 million patients with CVD, 13 and 11 million, have stroke and coronary heart disease, respectively. Those suggest the necessity for regular screening of circulating glucose and LDL-C concentrations to prevent the stroke and MI. Second, individuals with IFG and/or borderline high LDL-C at a first assessment should institute preventive measures, such as appropriate exercise and diet and stopping smoking, in an attempt to return their FBG and LDL-C concentrations to normal. Third, because of limited evidence, the existing guidelines for cholesterol treatment (62, 63) focus on the management of high LDL-C and neglect the management of borderline high concentrations, and guidelines for the management of diabetes (64) focus on the management of IFG and diabetes, but neglect the management of individuals with both IFG and borderline high LDL-C. The present findings provide a rationale for the combined management of IFG and borderline high LDL-C to prevent CVD.

The strengths of the present study were its large sample size, the long follow-up period, and the adjustment of the analyses for potentially confounding factors. In addition, we have characterized the relationship not only of IFG in combination with borderline high LDL-C at a single time point, but also through long-term follow-up. However, we also acknowledge several limitations. First, we focused on the outcomes of changes in the serum FBG and LDL-C concentrations and were unable to determine the exact causes of these changes, but we were able to be confident that they were not drug-induced, because the participants were not taking either glucose- or lipid-lowering drugs. Second, although we adjusted for a variety of factors, residual confounders may still have existed. Finally, all the participants were employees or retirees of the Kailuan Group and most were men (76.37%); therefore, the generalizability of the present findings is relatively limited. However, the Kailuan study involves a large number of participants with diverse occupations; therefore, the findings should still be instructive.

In conclusion, we have shown that individuals with both IFG and borderline high LDL-C are at higher risk of CVD than those with both normality. Furthermore, changes in IFG and borderline high LDL-C status within these ranges are associated with changes in the risk of CVD. These findings highlight the importance of changes in IFG and borderline high LDL-C status for the prevention and control of CVD.

## Data Availability Statement

The datasets used and/or analyzed during the present study are available from the corresponding author on reasonable request.

## Ethics Statement

The study was performed according to the guidelines of the Declaration of Helsinki and was approved by the Ethics Committee of Kailuan General Hospital (approval number: 2006-05). All the participants agreed to take part in the study and provided their written informed consent.

## Author Contributions

XW, Y-FZ, ZH, XY, and ZKC were responsible for drafting the manuscript and analyzing the data. SW and YC were responsible for designing the study. ZKC, WL, ZFC, YL, WF, GC, and WW were responsible for developing the first draft of the manuscript. All authors contributed to the writing of the manuscript and read and approved the final manuscript.

## Conflict of Interest

The authors declare that the research was conducted in the absence of any commercial or financial relationships that could be construed as a potential conflict of interest.

## Publisher’s Note

All claims expressed in this article are solely those of the authors and do not necessarily represent those of their affiliated organizations, or those of the publisher, the editors and the reviewers. Any product that may be evaluated in this article, or claim that may be made by its manufacturer, is not guaranteed or endorsed by the publisher.
