# Supplementary material for: Changes in Impaired Fasting Glucose and Borderline High Low-Density Lipoprotein-Cholesterol Status Alter the Risk of Cardiovascular Disease: A 9-Year Prospective Cohort Study
Source: Front Cardiovasc Med. 2022 Jun 21;9:882984. doi: 10.3389/fcvm.2022.882984 (PMC9253372; doi:10.3389/fcvm.2022.882984)
Supplement: Supplementary file 1 [file Data_Sheet_1.doc]

**Supplementary Figure 1.** Timeline of the study design.

Abbreviation: FBG: fasting blood glucose; IFG: impaired fasting glucose; LDL-C: low-density lipoprotein cholesterol.

IFG and borderline high LDL-C assessment period

Follow-up for outcome ascertainment

Year Year Year

2006 2008 2010

End of follow-up

(31 December 2019)

**Supplementary Table 1.** Missing covariate rate

| Variables | Total | Missing | Rate (%) |
| --- | --- | --- | --- |
| SBP (mmHg) | 36537 | 79 | 0.22% |
| DBP (mmHg) | 36537 | 74 | 0.20% |
| BMI (kg/m^2^) | 36537 | 228 | 0.62% |
| FBG (mmol/L) | 36537 | 0 | 0 |
| LDL-C (mmol/L) | 36537 | 0 | 0 |
| HDL-C (mmol/L) | 36537 | 492 | 1.34% |
| eGFR(ml/min/1.73m^2^) | 36537 | 102 | 0.28% |
| Current drinker, N (%) | 36537 | 1 | 0.003% |
| Current smoker, N (%) | 36537 | 1 | 0.003% |
| Senior college, N (%) | 36537 | 6 | 0.016% |
| Physical exercise, N (%) | 36537 | 3 | 0.008% |

Notes: BMI, body mass index; SBP, systolic blood pressure; DBP, diastolic blood pressure; HDL-C, high-density lipoprotein-cholesterol; LDL-C, low-density lipoprotein-cholesterol; FBG, fasting blood glucose, and eGFR, estimated glomerular filtration rate.

**Supplementary Table 2.** HR (95% CI) of CVD by change of IFG and borderline high LDL-C status in with stratification.

| Baseline | Both LDL-C and FBG Normal | | |  | IFG and (or) Borderline High LDL-C | | | *P* for Trend |
| --- | --- | --- | --- | --- | --- | --- | --- | --- |
| Follow-up | Both FBG and LDL-C Normal | IFG or Borderline High LDL-C | IFG and Borderline High LDL-C |  | Both FBG and LDL-C Normal | IFG or Borderline High LDL-C | IFG and Borderline High LDL-C |
| **Age（*P* for Interaction 0.57）** | | |  | |  |  |  |  |
| <45 | 1.00 | 1.13(0.71,1.81) | 1.21(0.29,5.02) | | 0.76(0.33,1.76) | 1.10(0.44,2.79) | 2.76(0.76,10.00) | 0.66 |
| ≥45 | 1.00 | 1.06(0.94,1.19) | 1.26(0.98,1.62) | | 1.02(0.84,1.24) | 1.24(1.00,1.52) | 1.42(1.00,2.10) | 0.03 |
| **Sex（*P* for Interaction 0.23）** | | |  | |  |  |  |  |
| Female | 1.00 | 1.36(0.95,1.94) | 0.92(0.42,2.03) | | 1.05(0.55,1.98) | 1.20(0.60,2.40) | 1.41(0.41,4.84) | 0.50 |
| Male | 1.00 | 1.03(0.92,1.17) | 1.30(1.00,1.68) | | 1.02(0.84,1.25) | 1.23(1.00,1.53) | 1.49(1.00,2.21) | 0.03 |
| **Hypertension（*P* for Interaction 0.51）** | | |  | |  |  |  |  |
| Yes | 1.00 | 1.06(0.91,1.23) | 1.24(0.89,1.71) | | 1.03(0.80,1.34) | 1.24(0.94,1.63) | 1.97(1.24,3.12) | 0.04 |
| No | 1.00 | 1.05(0.88,1.26) | 1.30(0.89,1.90) | | 0.94(0.73,1.25) | 0.92(0.48,1.76) | 1.21(0.89,1.64) | 0.48 |
| **Anti-hypertension drugs （*P* for Interaction 0.86）** | | | | |  |  |  |  |
| Yes | 1.00 | 1.10(0.85,1.42) | 1.43(0.87,235) | | 1.00(0.63,1.56) | 1.41(0.60,3.34) | 1.57(1.02,2.41) | 0.07 |
| No | 1.00 | 1.05(0.92,1.19) | 1.21(0.91,1.61) | | 1.00(0.80,1.22) | 1.14(0.90,1.43) | 1.49(1.00,2.26) | 0.16 |
| **Smoking status (*P* for Interaction 0.11)** | | | | |  |  |  |  |
| Yes | 1.00 | 1.16(0.97,1.38) | 1.10(0.73,1.65) | | 0.80(0.58,1.09) | 1.30(0.96,1.76) | 1.35(0.77,2.34) | 0.28 |
| No | 1.00 | 1.00(0.85,1.16) | 1.38(1.01,1.88) | | 1.14(0.89,1.46) | 1.13(0.85,1.49) | 1.57(0.94,2.62) | 0.09 |
| **Drinking status (*P* for Interaction 0.09)** | | | | |  |  |  |  |
| Yes | 1.00 | 1.23(1.02,1.47) | 1.35(0.92,1.96) | | 0.86(0.62,1.19) | 1.36(0.99,1.86) | 1.70(1.02,2.82) | 0.06 |
| No | 1.00 | 0.97(0.83,1.12) | 1.22(0.88,1.70) | | 1.07(0.84,1.36) | 1.11(0.84,1.46) | 1.23(0.69,2.18) | 0.32 |
| **Physical activity** **(*P* for Interaction 0.25)** | | | | |  |  |  |  |
| Yes | 1.00 | 0.98(0.74,1.30) | 1.51(0.94,2.43) | | 0.80(0.50,1.26) | 0.89(0.55,1.44) | 2.02(1.05,3.90) | 0.70 |
| No | 1.00 | 1.08(0.95,1.23) | 1.17(0.87,4.57) | | 1.03(0.83,1.27) | 1.23(0.98,1.96) | 1.29(1.03,1.62) | 0.05 |

Adjusted hazard ratio with 95% confidence interval were noted.

Adjusted for age, sex, obesity, smoking status, drinking status, physical exercise status, educational level, hypertension, use of anti-hypertensive drugs, HDL-C, and eGFR in 2010, LDL-C and FBG in 2006.

Abbreviations: LDL-C, low-density lipoprotein-cholesterol; HDL-C, high-density lipoprotein-cholesterol; FBG, fasting blood glucose; IFG, impaired fasting glucose; CVD: cardiovascular disease; HR, hazard ratio; CI, confidence interval; eGFR: estimated glomerular filtration rate.

**Supplementary Table 3.** Sensitivity analysis of HR (95% CI) of CVD by change of IFG and borderline high LDL-C status

| Baseline | Both LDL-C and FBG Normal | | |  | | IFG and (or) Borderline High LDL-C | | | *P* for Trend |
| --- | --- | --- | --- | --- | --- | --- | --- | --- | --- |
| Follow-up | Both FBG and LDL-C Normal | IFG or Borderline High LDL-C | IFG and Borderline High LDL-C |  | | Both FBG and LDL-C Normal | IFG or Borderline High LDL-C | IFG and Borderline High LDL-C |
| Sensitivity analysis 1 | |  |  | |  | |  |  |  |
| HR (95% CI) | 1.00 | 1.04(0.92,1.17) | 1.14(0.87,1.49) | | 0.97(0.80,1.19) | | 1.11(1.07,1.50) | 1.24(1.02,1.34) | 0.02 |
| Sensitivity analysis 2 | |  |  | |  | |  |  |  |
| HR (95% CI) | 1.00 | 1.06(0.95,1.20) | 1.32(1.03,1.69) | | 1.01(0.83,1.23) | | 1.24(1.00,1.52) | 1.51(1.03,2.22) | 0.02 |
| Sensitivity analysis 3 | |  |  | |  | |  |  |  |
| HR (95% CI) | 1.00 | 1.06(0.94,1.19) | 1.26(0.98,1.61) | | 1.00(0.82,1.20) | | 1.23(1.00,1.51) | 1.48(1.02,2.15) | 0.04 |
| Sensitivity analysis 4 | |  |  | |  | |  |  |  |
| HR (95% CI) | 1.00 | 1.08(0.96,1.22) | 1.31(1.02,1.68) | | 0.98(0.81,1.19) | | 1.25(1.02,1.54) | 1.42(1.01,2.07) | 0.03 |
| Sensitivity analysis 5 | |  |  | |  | |  |  |  |
| HR (95% CI) | 1.00 | 1.08(0.96,1.22) | 1.31(1.02,1.68) | | 0.98(0.81,1.19) | | 1.25(1.02,1.54) | 1.42(1.00,2.07) | 0.05 |

Sensitivity analysis 1: Excluding outcome events within the first year of follow-up (n=257),

Sensitivity analysis 2: Excluding missing covariable (n=859).

Sensitivity analysis 3: adjusted for age, sex, overweight, smoking status, drinking status, physical exercise status, educational level, hypertension, use of anti-hypertensive drugs, HDL-C, and eGFR in 2010, and LDL-C, FBG in 2006.

Sensitivity analysis 4: adjusted for age, sex, obesity, smoking status, drinking status, physical exercise status, educational level, hypertension, use of anti-hypertensive drugs, LDL-C, FBG, HDL-C, and eGFR in 2006.

Sensitivity analysis 5: adjusted for age, sex, obesity, smoking status, drinking status, physical exercise status, educational level, hypertension, use of anti-hypertensive drugs, LDL-C, FBG, HDL-C, and eGFR in 2006 and 2010.

Abbreviations: HDL-C, high-density lipoprotein-cholesterol; LDL-C, low-density lipoprotein-cholesterol; FBG, fasting blood glucose; IFG, impaired fasting glucose; CVD, cardiovascular disease; HR, hazard ratio; CI, confidence interval; eGFR: estimated glomerular filtration rate.

**Supplementary Table 4.** Sensitivity analysis of HR (95% CI) of CVD by change of IFG and borderline high LDL-C status

| Baseline | Both LDL-C and FBG Normal | |  | IFG and (or) Borderline High LDL-C | | *P* for Trend |
| --- | --- | --- | --- | --- | --- | --- |
| Follow-up | Both LDL-C and FBG Normal | IFG and (or) Borderline High LDL-C |  | Both LDL-C and FBG Normal | IFG and (or) Borderline High LDL-C |
| **Cardiovascular Disease** | |  |  |  |  |  |
| Case/Total | 861/21091 | 521/9288 |  | 177/3605 | 194/2553 |  |
| Incidence Rate* | 4.71 | 6.58 |  | 5.74 | 9.12 |  |
| HR (95% CI) | 1.00 | 1.08(0.97,1.21) |  | 1.00(0.81,1.22) | 1.25(1.02,1.52) | 0.04 |
|  |  |  |  |  |  |  |
| **Myocardial infarction** | |  |  |  |  |  |
| Case/Total | 161/21091 | 98/9301 |  | 32/3636 | 43/2556 |  |
| Incidence Rate* | 0.87 | 1.21 |  | 1.02 | 1.96 |  |
| HR (95% CI) | 1.00 | 1.10(0.85,1.42) |  | 0.90(0.57,1.39) | 1.37(0.89,2.11) | 0.25 |
|  |  |  |  |  |  |  |
| **Stroke** |  |  |  |  |  |  |
| Case/Total | 700/21091 | 423/9288 |  | 145/3605 | 151/2553 |  |
| Incidence Rate* | 3.82 | 5.31 |  | 4.68 | 7.02 |  |
| HR (95% CI) | 1.00 | 1.08(0.95,1.22) |  | 1.01(0.82,1.25) | 1.20(0.96,1.50) | 0.12 |
|  |  |  |  |  |  |  |
| **Ischemic Stroke** | |  |  |  |  |  |
| Case/Total | 629/21091 | 375/9288 |  | 132/3605 | 136/2553 |  |
| Incidence Rate* | 3.43 | 4.70 |  | 4.25 | 6.31 |  |
| HR (95% CI) | 1.00 | 1.09(0.96,1.24) |  | 1.01(0.82,1.25) | 1.22(0.98,1.51) | 0.08 |
|  |  |  |  |  |  |  |
| **Hemorrhagic Stroke** | |  |  |  |  |  |
| Case/Total | 107/21091 | 58/9288 |  | 19/3605 | 16/2553 |  |
| Incidence Rate* | 0.58 | 0.72 |  | 0.60 | 0.72 |  |
| HR (95% CI) | 1.00 | 1.22(1.08,1.38) |  | 1.01(0.82,1.25) | 1.35(1.09,1.68) | <0.01 |

*Case per 1000 person-years

Model adjusted for age, sex obesity, smoking status, drinking status, physical exercise status, educational level, hypertension, use of anti-hypertensive drugs, HDL-C, eGFR in 2010, LDL-C, and FBG in 2006.

Abbreviations: HDL-C, high-density lipoprotein; LDL-C, low-density lipoprotein cholesterol; FBG, fasting blood glucose; IFG, impaired fasting glucose; CVD, cardiovascular disease; HR, hazard ratio; CI, confidence interval
